# Age-Related Macular Degeneration: Epidemiology, Pathophysiology, Diagnosis, and Treatment

**DOI:** 10.7759/cureus.29583

**Published:** 2022-09-26

**Authors:** Hrishikesh Vyawahare, Pranaykumar Shinde

**Affiliations:** 1 Ophthalmology, Jawaharlal Nehru Medical College, Datta Meghe Institute of Medical Sciences, Wardha, IND

**Keywords:** age-related macular degeneration, homeostasis, proteostasis, oxidative stress, retinal pigment epithelium (rpe), neuroinflammation

## Abstract

The greatest global root of irremediable amaurosis in the venerable is age-related macular degeneration (AMD), a complex eye condition. Clinically, AMD is characterized as being in an early stage to late stage and initially affects the macula, which is the center of the retina (advanced AMD). Age-related cellular and metabolic imbalance are made worse by the creation of excessive amounts of free radical species, which causes mitochondrial malfunction. As a result, in AMD-affected eyes, the deprivation of melanocytes, confection, and eventually atrophy within the retinal tissue are caused by the continued proliferation of oxidative stress caused by systemic antioxidant capacity depletion.

In the urbanized, industrialized world, age-related macular degeneration (AMD) is one of the major causes of central vision loss in the older age group. Although several causes and mechanisms for the dysfunction and degeneration of the retinal pigment epithelium (RPE) have previously been identified, the condition’s symptoms are still not fully understood. Etiopathogenesis is still not entirely understood. As a result, the RPE fails, allowing an accumulation of aberrant misfolded proteins, due to the loss of anatomical control over oppression, altered homeostasis, dysfunctional lipid homeostasis, and failure of mitochondria.

Due to the multitude of interconnected processes, numerous complicated therapy combinations will probably be the best option to deliver the best visual outcomes; these combinations will vary depending on the kind and degree of the condition being treated. Undoubtedly, this will lead to the development of customized preventative medications and, hopefully, the revelation of a potential cure. All the mechanisms involved in the etiology of AMD should be continuously probed to create covariates for other contemporaneous or future problems.

## Introduction and background

Age-related macular degeneration (AMD) affects one in eight people 60 years of age or older and is the most common cause of irreversible blindness in older persons in developed countries. According to thorough estimates, 200 million people worldwide are estimated to have AMD, and by 2040, this number is projected to rise to close to 300 million. By 2050, 5.4 million Americans are anticipated to be affected by rising patterns similar to this one. Despite the fact that the majority of these people are of Caucasian ancestry, intervening dry AMD and wet choroidal vasculopathy seem to occur more often in Asian and African American populations. The demographic shift brought on by an aging global population can be used to explain the expected increase in the prevalence of adults suffering from non-communicable eye disorders such as AMD. Due to its chronic character, which necessitates consistent long-term management, AMD has become and will remain a public health concern for both high- and low-income countries, with significant socioeconomic ramifications and increases in healthcare costs [1].

The macula is a 5.5-mm-diameter circular patch with a center that is 17 degrees or 4-5 mm temporal and 0.53-0.8 mm inferior to the center of the optic disc. In the human eye, a small center pit called the fovea centralis is made up of several closely spaced cones. For acute center vision, we can thank the fovea. The parafoveal belt and the perifoveal outer zone encircle the fovea. When a person is over 55, macular degeneration is the most common factor contributing to severe, permanent vision loss. The macula deteriorates, which causes it. Age-related macular degeneration (AMD) is a common name for the condition since it occurs as a person ages [2]. The most common reason for elderly people in industrialized nations to lose their vision is age-related macular degeneration (AMD). Geographic atrophy (GA) is one early and non-exudative symptom of AMD that has not yet received disease-specific treatment, despite the fact that anti-vascular endothelial growth factor (VEGF) therapy has significantly improved outcomes.

The assessment of visual impairment linked to AMD manifestations requires the establishment of valid functional endpoints in addition to the structural examination of alterations. The most often utilized functional endpoint, best-corrected visual acuity (BCVA), only assesses the photopic function of the central retina and is insensitive to assess therapeutic advantages outside of the fovea [3].

Neovascular age-related macular degeneration (nAMD), one of the most common causes of blindness, affects more than 200 million people worldwide. It is projected that its frequency will increase due to the aging population in many countries [4]. AMD is a retinal disorder that often affects the macula and gradually robs sufferers of their ability to see well in the center of their field of vision. Early-stage AMD is primarily characterized by clinical symptoms such as drusen and alterations to the retinal pigment epithelium. The two primary kinds of late-stage AMD that might exist are neovascular (also known as wet or exudative) and non-neovascular (also referred to as atrophic, dry, or non-exudative) AMD. The quality of life of those who are affected is substantially damaged by AMD’s progression into its late stages, which results in central vision loss, severe and irreversible visual impairment, and legal blindness [5].

There are several AMD symptoms that can be observed, some of which include geographic atrophy and an accumulation of drusen beneath the retinal pigment epithelium. AMD varieties are categorized as exudative (wet) and non-exudative (dry). Exudative AMD demonstrates choroidal neovascularization (CNV), which sets it apart from the dry, often slower progressing variant of the disease.

Although substantial research has been done to understand the pathogenesis of AMD’s macular symptoms, the retina’s periphery still needs to be studied extensively. Despite research showing that peripheral pigmentary, drusenoid, and atrophic retinal abnormalities are, in fact, common in AMD patients, the impact of these discoveries on AMD is not entirely understood. Thanks to recent developments in imaging technology, we can now more extensively analyze peripheral retinal data. The identification of peripheral retinal manifestations using ultra-widefield (UWF) fundus imaging has led to the development of a special UWF image-based grid grading system [6].

## Review

Epidemiology

AMD prevalence varies greatly by ethnicity, with non-Hispanic White Europeans carrying the majority of the disease burden. We use the terms race and ethnicity in this review, as did the authors of the original study. Since participants are evaluated diversely in each research, we do not attempt to standardize the names. Notwithstanding a little fluctuation, there is still a significant chronic burden among Hispanics (10.4%), Africans (7.5%), and Asians (7.4%). Even yet, other researchers have estimated a lower disease burden in the United States, with non-Hispanic White Europeans having the highest frequency at about 7.3% [7].

In a population-based cohort study that combined data from three population-based cohort studies (mean age: 60.1-65.7 years), age-adjusted dry AMD incidence ranged from 0.3% to 0.4%, with follow-up times ranging from 4.8 to 6.5 years; increased incidence was linked to late-stage AMD disease at baseline. In a North American study (N = 3,549), the incidence of dry AMD was also associated with baseline drusen status, with the 10-year incidence risk of dry AMD diagnosis increasing from 9.9% in patients with large bilateral drusen at baseline (n = 241) to 44.3% in patients with large bilateral drusen and RPE changes at baseline (n = 636) and 53.9% in patients with late AMD (dry or wet) in one eye at baseline (n = 390) [8]. Table 1 presents the stages of AMD and their characteristics.

**Table 1:** Age-related macular degeneration and its stages AMD: age-related macular degeneration

Stages	Characteristics
No AMD	No drusen, no AMD pigmentary abnormalities, and normal dark adaptations
Subclinical AMD	No drusen or small drusen, no AMD pigmentary abnormalities, and impaired dark adaptations
Early AMD	Small or medium drusen, AMD pigmentary abnormalities, and impaired dark adaptations
Intermediate AMD	One large druse, any AMD pigmentary abnormalities, and impaired dark adaptations
Advanced AMD	Geographic atrophy and neovascular AMD

Pathophysiology

It may result from a number of modifications brought on by the aging of RPE cells. In the macular region, the delicate enzyme balance of the extracellular matrix (ECM) is broken by aging RPE cells. Senescent RPE cells trigger the immune system’s production of VEGF. Blood vessels are created by the Bruch membrane’s calcification, rupturing, and phagocytosis, and AMD is ultimately caused by these processes [2]. When circumstances threaten the equilibrium of cells and tissues, the body’s cells respond by inducing inflammation. Cell-associated and soluble pattern recognition receptors, including Toll-like receptors, heterodimeric receptors, and complement components, prompt intricate cellular cascades by recognizing or detecting multifaceted pathogen- and damage-associated molecular patterns, respectively. The eye’s RPE cells face additional challenges, including preserving the health of the Purkinje cells above them, insulating the retina from excessive light, cultivating the blood-retinal barrier with the vascular endothelium, and adaptive defense of the central retina (macula). The autophagic breakdown of used-up photoreceptor outer segment (POS) tips, also known as heterophagy, is one of the primary roles of RPE. Continuous consumption of POS by non-dividing, aged RPE cells causes lysosomes to accumulate lipofuscin, an autofluorescent, degradable compound that prevents autophagy by impeding the activity of lysosomal enzymes. This process combines oxidative stress with retinal inflammation [9].

The complicated etiology of AMD has been connected to cellular, biochemical, and molecular processes and is impacted by a number of variables, including both environmental influences and genetic predisposition, despite the fact that the pathogenesis of AMD is presently not fully known. The known genetic risk factors evaluated together assert that oxidative stress, lipid metabolism, extracellular matrix biology, inflammation, liberalization of the complement cascade, and other immunological responses are involved in the pathophysiology of the illness.

Early AMD culminates in aberrant RPE pigment distribution in the macula, and between the inner collagenous layer of Bruch’s membrane and the RPE’s basal lamina, in the retina, a variety of proteins and drusen-containing lipids are garnered. Although early AMD is typically asymptomatic, it can produce a little loss in visual acuity and function that delays the onset of night blindness [10].

A middle stage of dry AMD and atrophy, or the loss of the retina's outer layers, are linked to RPE loss. In the intermediate version, the patient may also express concerns about decreased reading speed, sensitivity to contrast, and trouble responding to changes in lighting conditions. A patient must have both atrophy and a significant loss of central vision in order to be diagnosed with the advanced type of dry AMD. Peripheral visual acuity is preserved despite the nonexudative AMD’s form. GA is the condition in which the RPE atrophy spreads to broader regions in the non-exudative AMD area. When CNV develops, GA, which is described as bilateral, but not symmetrical, might hasten the loss of vision. Identification of individuals who will eventually acquire an advanced form of AMD is very important, as stated in Section 2 by the Age-Related Eye Disease Study Group. Non-exudative AMD is defined by the RPE atrophy score, which is comprised of the loss of visual acuity, loss of visual field, and loss of photoreceptor cells [11]. Oxidative stress plays a significant part in how AMD progresses pathophysiological. Oxygen-free radical generation, which finally results in cell death via a variety of processes, is the primary characteristic of cellular aging. Vitamins C and E, carotenoids, lutein, and zeaxanthin all play a role in the retina’s healthy defense against oxidative processes. Additionally, the presence of a significant structural lipid at the level of cones called docosahexaenoic acid (DHA) affects membrane permeability and inhibits the growth of new blood vessels [5].

Several pathogenic mechanisms can cause AMD at the molecular and biochemical levels, according to our current understanding of the disease. These consist of oxidative damage, aberrant lipid metabolism, apoptosis, structural changes to outer photoreceptor segments, RPE ion channel malfunction, immune system changes, and abnormalities of the extracellular matrix. There are differences in the extracellular matrix composition across the retina, and any modification, such as depletion, synthesis, or enhanced breakdown and waste, can result in retinal alterations linked to AMD [12].

Today, there is still a significant therapeutic difference between dry AMD and wet AMD. Wet AMD can be treated right now, on the one hand. Wet AMD is now mostly treated with monthly intravitreal injections of anti-VEGF medications, which can considerably lower the risk of severe vision loss. Only a small population, though, may reasonably benefit from this strategy. In this sense, a novel therapy for wet AMD is also necessary. However, despite considerable advancements in etiology, there is still no authorized medicine or effective treatment for dry AMD. Therefore, finding a successful treatment for dry AMD is more critical than it is for wet AMD [13].

Neovascular age-related macular degeneration

Degeneration is linked to aging and was the third most common cause of blindness in 2015. Recently, its prevalence has significantly grown as birth rates have drastically fallen. Age, smoking, cataract surgery, BMI, vascular diseases, hypertension, fibrinogen, atherosclerosis, high-density lipoprotein cholesterol (HDL-C), and the blue light from smart devices are just a few of the factors that might affect the development of AMD.

Corticosteroids and other nonsteroidal anti-inflammatory drugs (NSAIDs) are very effective at reducing angiogenesis and inflammation in AMD, but because of their numerous side effects, including preeclampsia, insulin sensitivity, chronic fatigue, tearfulness, uveitis, skin thinning, cataracts, glaucoma, and gastric ulceration, they are only used in combination therapies with photodynamic therapy or anti-VEGF drugs. Additionally, anti-VEGF medication has rarely been examined to be the most effective form of long-term NSAID treatment for AMD.

Although AMD is increasingly common as people age, if we can stop AMD from advancing to more severe forms, we can reduce the prevalence of blindness in the general population [14]. Age-related macular degeneration (AMD) with neovascularization is a prevalent cause of vision loss worldwide. Over 200 million individuals worldwide are affected by neovascular age-related macular degeneration (AMD), one of the most widespread causes of blindness. With the aging population in many nations, it is anticipated that its prevalence will rise. Around the world, nAMD is still a major factor in vision loss.

Although the development of anti-VEGF medications gave people with AMD fresh hope, the necessity of long-term therapy and the frequency of injections place a considerable financial and logistical burden on both individuals and the healthcare system [15]. The pathogenesis of AMD includes oxidative stress and retinal inflammatory pathways. Wet AMD and dry AMD are the two main categories of the illness. After the development of new blood vessels in the retina and subretinal region, dry AMD may evolve into wet AMD (nAMD). Following advancement, these arteries result in bleeding, serum leakage, fluid retention, visual distortion, and central vision loss [16]. As a result, mitochondrial dysfunction, altered proteostasis, altered lipid homeostasis, and lack of cellular control of oxidative stress combine to create an internal feedback loop that leads to the failure of the RPE and the accumulation of abnormal misfolded proteins and abnormal lipids that will eventually form drusen. Over time, aberrant drusen material is more likely to accumulate due to insufficient antioxidant defenses, deficiencies in autophagy systems, and dysregulation of the extracellular matrix (ECM). Once the subretinal region becomes chronically inflamed, the drusen operate as inflammatory centers that recruit macrophages and microglia to the area. The complement system plays a significant role in this process [17]. Normal outcomes of the non-neovascular form include progressive vision deterioration, geographic atrophy, and slow but steady degradation of the outer retina. As a result of choroidal neovascular membrane development, exudation, and fibrosis, nAMD causes an abrupt loss of vision [18].

Neovascular age-related macular degeneration (AMD), sometimes known as “wet” AMD, is an advanced type of AMD characterized by choroidal neovascularization (CNV), in which newly created blood vessels leak into the retina, producing distortion and fast loss of vision [19]. According to scientific literature, prolonged exposure to light can harm surface tissues, including the skin and eyes. This is why the development of AMD may potentially be accompanied by the damage caused by sunshine exposure [20]. Due to photoreceptor damage brought on by exudation processes, CNV is a significant contributor to vision loss in nAMD.

Optical coherence tomography (OCT) is currently the gold standard for evaluating the existence of CNV and exudation, even if fluorescein angiography (FA) has historically been the gold standard for characterizing and identifying CNV lesions [21]. It is thought that oxidation, inflammation, and angiogenesis are all exacerbated by the external pressures that come with age. Events that are pathologic take place when external pressures overcome equilibrium. Although anti-angiogenic therapies that block the bioactivity of vascular endothelial growth factor (anti-VEGF) have changed results in nAMD, over 50% of patients still lose their eyesight after therapy [22]. Only the exudative types of the condition are now treatable, and the medication used does so by blocking vascular endothelial growth factor (VEGF). Subretinal fibrosis and a fibrotic scar may develop from untreated exudative AMD [23]. Patients with intermediate AMD who are at high risk of developing late nAMD should be properly monitored as soon as macular neovascularization vessels are identified [24].

Diagnosis

Fluorescein angiography, optical coherence tomography, indocyanine green angiography, artificial intelligence (AI), and fundus imaging are some of the methods designed to detect AMD. These modalities are essential for the diagnosis of AMD, and each has perks. Fundus imaging methods include color photography, monochromatic photography, automatic fluorescence imaging, and fundus angiography, which are useful for AMD diagnosis [2]. To visualize the neovascular complex, OCT angiography (OCT-A) is thought to be useful if it is accessible. FA can be performed to see the leakage from the lesion if OCT-A is unavailable. However, it is no longer considered to be a necessary approach for diagnosing all instances of AMD [25]. AMD develops from an early, middle, and advanced dry form to a final, more severe type that shows up as geographic atrophy and choroidal neovascularization. The transition to nAMD is characterized as progression to advanced dry AMD, and the presence of geographic atrophy is known as progression to AMD-related exudation. Predictions of AMD development may enable prompt surveillance, early discovery, and treatment, improving visual results [26].

Treatment

Current research in dry AMD is examining a number of therapy options with a wide variety of targets. Investigated methods to slow the rate of illness progression include antioxidative medications, complement cascade inhibitors, neuroprotective compounds, visual cycle inhibitors, gene therapy, and cell-based therapeutics [27]. Gene therapy products may now be administered intravitreally to significantly lessen the burden of treatment and enhance visual outcomes. The finest medication delivery solution for treating AMD is also provided by the most recent advancements in nanotherapy [28]. Focused photocoagulation was the initial form of treatment for diabetic macular edema (DME), which later gave way to injections of anti-VEGF [29]. Several pathogenic variables, including inflammation and retinal cell death, can be prevented by metformin (MET), according to recent research. It can also control lipid metabolism and prevent the formation of CNV [30]. Poor patient adherence and delayed nAMD treatment initiation can both lead to less-than-ideal results, emphasizing the necessity for management strategies that integrate prompt and efficient therapy at intervals tailored to the needs of each patient [31]. More dietary carotenoids were linked to a lower chance of developing late-onset AMD, according to epidemiology studies, whereas higher levels of macular pigment were linked to a substantial improvement in visual function in AMD patients [32]. In degenerative disorders such as dry AMD, a number of implanted prosthetic devices are being developed and researched to restore eyesight [33]. Age-related macular degeneration with neovascularization has a significantly altered prognosis due to the use of anti-vascular endothelial growth factor (VEGF) therapies (nAMD). Progressive dry (non-neovascular) AMD has no proven effective treatment, unlike wet AMD. However, stem cell-based treatments, a component of regenerative medicine, have produced encouraging outcomes for degenerative retinal conditions such as AMD.

## Conclusions

Age-related macular degeneration with neovascularization can have catastrophic effects on one’s vision, society, and finances. There are many different ways to treat it, and its pathophysiology is complicated. The anti-VEGF route has traditionally been the focus of therapies, which have produced superior outcomes than earlier laser, sham, and surgical procedures. The more structured therapy alternatives, nevertheless, can be constrained by management requirements, cost considerations, and patient compliance issues. In terms of visual outcomes and quality of life, AMD continues to have a significant influence on the global population despite the positive findings. While we wait for new medications that will be able to improve the clinical course of AMD even more than we can accomplish right now, implementing preventative techniques may be an option. For a thorough knowledge of this blinding condition, it is important to define the molecular, physiological, and pathological functions of each of the components in AMD development and progression. An interesting possibility for improved comprehension and fresh perspectives on retinal illness is presented by the application of deep learning, sophisticated feature interrogation techniques, and radiomics characterization. Precision medicine and individualized therapy are becoming more possible because of the field of computational imaging biomarker identification and research in AMD and diabetic eye disease.

## References

[1] Deng Y, Qiao L, Du M, Qu C, Wan L, Li J, Huang L (2022). Age-related macular degeneration: epidemiology, genetics, pathophysiology, diagnosis, and targeted therapy. Genes Dis.

[2] von der Emde L, Pfau M, Holz FG (2021). AI-based structure-function correlation in age-related macular degeneration. Eye (Lond).

[3] Tan CS, Ngo WK, Chay IW, Ting DS, Sadda SR (2022). Neovascular age-related macular degeneration (nAMD): a review of emerging treatment options. Clin Ophthalmol.

[4] Di Carlo E, Augustin AJ (2021). Prevention of the onset of age-related macular degeneration. J Clin Med.

[5] Pivovar A, Oellers P (2021). Peripheral manifestations in age related macular degeneration: a review of imaging and findings. J Clin Med.

[6] Pennington KL, DeAngelis MM (2016). Epidemiology of age-related macular degeneration (AMD): associations with cardiovascular disease phenotypes and lipid factors. Eye Vis (Lond).

[7] Schultz NM, Bhardwaj S, Barclay C, Gaspar L, Schwartz J (2021). Global burden of dry age-related macular degeneration: a targeted literature review. Clin Ther.

[8] Kauppinen A, Paterno JJ, Blasiak J, Salminen A, Kaarniranta K (2016). Inflammation and its role in age-related macular degeneration. Cell Mol Life Sci.

[9] Berber P, Grassmann F, Kiel C, Weber BH (2017). An eye on age-related macular degeneration: the role of microRNAs in disease pathology. Mol Diagn Ther.

[10] Fernandes AR, Zielińska A, Sanchez-Lopez E (2022). Exudative versus nonexudative age-related macular degeneration: physiopathology and treatment options. Int J Mol Sci.

[11] Al Gwairi O, Thach L, Zheng W, Osman N, Little PJ (2016). Cellular and molecular pathology of age-related macular degeneration: potential role for proteoglycans. J Ophthalmol.

[12] Qin S, Dong N, Yang M, Wang J, Feng X, Wang Y (2021). Complement inhibitors in age-related macular degeneration: a potential therapeutic option. J Immunol Res.

[13] Cho YK, Park DH, Jeon IC (2021). Medication trends for age-related macular degeneration. Int J Mol Sci.

[14] Nguyen V, Barthelmes D, Gillies MC (2021). Neovascular age-related macular degeneration: a review of findings from the real-world Fight Retinal Blindness! registry. Clin Exp Ophthalmol.

[15] Sarkar A, Junnuthula V, Dyawanapelly S (2021). Ocular therapeutics and molecular delivery strategies for neovascular age-related macular degeneration (nAMD). Int J Mol Sci.

[16] Garcia-Garcia J, Usategui-Martin R, Sanabria MR, Fernandez-Perez E, Telleria JJ, Coco-Martin RM (2022). Pathophysiology of age-related macular degeneration. Implications for treatment. Ophthalmic Res.

[17] Arepalli S, Kaiser PK (2021). Pipeline therapies for neovascular age related macular degeneration. Int J Retina Vitreous.

[18] Phan LT, Broadhead GK, Hong TH, Chang AA (2021). Predictors of visual acuity after treatment of neovascular age-related macular degeneration - current perspectives. Clin Ophthalmol.

[19] Kalra G, Kar SS, Sevgi DD, Madabhushi A, Srivastava SK, Ehlers JP (2021). Quantitative imaging biomarkers in age-related macular degeneration and diabetic eye disease: a step closer to precision medicine. J Pers Med.

[20] Ramshekar A, Wang H, Hartnett ME (2021). Regulation of Rac1 activation in choroidal endothelial cells: insights into mechanisms in age-related macular degeneration. Cells.

[21] Klettner A, Roider J (2021). Retinal pigment epithelium expressed toll-like receptors and their potential role in age-related macular degeneration. Int J Mol Sci.

[22] Flores R, Carneiro Â, Tenreiro S, Seabra MC (2021). Retinal progression biomarkers of early and intermediate age-related macular degeneration. Life (Basel).

[23] Kodjikian L, Parravano M, Clemens A (2021). Fluid as a critical biomarker in neovascular age-related macular degeneration management: literature review and consensus recommendations. Eye (Lond).

[24] Romond K, Alam M, Kravets S (2021). Imaging and artificial intelligence for progression of age-related macular degeneration. Exp Biol Med (Maywood).

[25] Cabral de Guimaraes TA, Daich Varela M, Georgiou M, Michaelides M (2022). Treatments for dry age-related macular degeneration: therapeutic avenues, clinical trials and future directions. Br J Ophthalmol.

[26] Yang B, Li G, Liu J, Li X, Zhang S, Sun F, Liu W (2021). Nanotechnology for age-related macular degeneration. Pharmaceutics.

[27] Muste JC, Russell MW, Singh RP (2021). Photobiomodulation therapy for age-related macular degeneration and diabetic retinopathy: a review. Clin Ophthalmol.

[28] Dang KR, Wu T, Hui YN, Du HJ (2021). Newly-found functions of metformin for the prevention and treatment of age-related macular degeneration. Int J Ophthalmol.

[29] Daien V, Finger RP, Talks JS (2021). Evolution of treatment paradigms in neovascular age-related macular degeneration: a review of real-world evidence. Br J Ophthalmol.

[30] Lem DW, Davey PG, Gierhart DL, Rosen RB (2021). A systematic review of carotenoids in the management of age-related macular degeneration. Antioxidants (Basel).

[31] Rubner R, Li KV, Canto-Soler MV (2022). Progress of clinical therapies for dry age-related macular degeneration. Int J Ophthalmol.

[32] Veritti D, Sarao V, Soppelsa V, Danese C, Chhablani J, Lanzetta P (2022). Managing neovascular age-related macular degeneration in clinical practice: systematic review, meta-analysis, and meta-regression. J Clin Med.

[33] Dehghan S, Mirshahi R, Shoae-Hassani A, Naseripour M (2022). Human-induced pluripotent stem cells-derived retinal pigmented epithelium, a new horizon for cells-based therapies for age-related macular degeneration. Stem Cell Res Ther.

